# Association between stroke and memory diseases: evidence from a prospective national cohort study in China

**DOI:** 10.3389/fneur.2025.1578200

**Published:** 2025-06-19

**Authors:** Kai Zhou, Jiehua Gan, Guomin Xie, Xiao Chen, Zhongyue Lv

**Affiliations:** Department of Neurology, Ningbo Medical Center Lihuili Hospital, Ningbo University, Ningbo, Zhejiang, China

**Keywords:** memory diseases, Alzheimer’s disease, brain atrophy, stroke, China Health and Retirement Longitudinal Study

## Abstract

**Background:**

Previous studies had shown stroke played an important role in the pathogenesis of memory diseases. Thus, this study investigated the correlation between stroke and memory diseases [Alzheimer’s disease (AD) and brain atrophy] and provided a new theoretical basis for the diagnosis of stroke disease.

**Methods:**

A total of 15,904 total participants were obtained based on the China Health and Retirement Longitudinal Study (CHARLS), including 322 stroke subjects and 15,582 control subjects. Stroke was outcome variable, after the questionnaire, the subjects were divided into stroke and control groups. Meanwhile, various categorical variables, such as memory diseases (AD, brain atrophy), gender and medical insurance were included in this study. The weighted chi-square test was used to analyze whether there were differences in covariates between stroke and control groups. In addition, the correlation between memory diseases and stroke were analyzed by weighted logistic regression. Receiver operating characteristic (ROCs) curves were used to assess the accuracy and reliability of the Model III.

**Results:**

Stroke and control subjects differed significantly in a variety of clinical characteristics, and variables such as healthy status, patient service and memory diseases were significantly correlated with stroke prevalence. According to the three models constructed in this study, memory diseases was significantly associated with stroke in all three models (Model I, odds ratio (OR) = 7.33, *p* < 0.001, 95% Confidence interval (CI) = 5.31–9.94; Model II, OR = 7.33, *p* < 0.001, 95% CI = 5.31–9.95; Model III, OR = 3.84, *p* < 0.001, 95% CI = 2.73–5.30). Weighted logistic regression analysis showed the stability of the relationship between memory diseases and stroke, further suggested the correlation between memory diseases and stroke. Finally, the area under curve (AUC) of 0.778 indicated that the prediction accuracy of Model III was better.

**Conclusion:**

According to the results in this study, there was a significant association between memory diseases and stroke. It is worthwhile to further study the mechanisms between stroke and memory diseases.

## 1 Introduction

Stroke involves acute disruptions in cerebral blood circulation due to factors causing arterial stenosis, blockage, or rupture, which clinically present as temporary or permanent neurological dysfunction (1). Globally, stroke ranks as the second leading cause of death and the third leading cause of disability, characterized by high incidence, high disability rates, and high mortality (2). Vascular factors, including large artery disease, cardioembolism, and small vessel disease, are the leading causes of ischemic stroke (3). Currently, the effective methods for acute recanalization in ischemic stroke include intravenous thrombolysis, intra-arterial thrombolysis, and mechanical thrombectomy (4). However, due to the limited therapeutic time window, only a small fraction of patients can undergo recanalization therapy (5). Most patients are left with sequelae such as hemiplegia, aphasia, and cognitive decline (6). Therefore, it is crucial to investigate stroke mechanisms and create early diagnostic and preventive strategies.

The risk of stroke is associated with factors such as hypertension, diabetes, high cholesterol, heart disease, smoking, alcohol consumption, dietary habits, being overweight, physical inactivity, and psychological factors (7–9), some of which are also risk factors for Alzheimer’s disease (AD) (10). Recent studies suggest that memory-related diseases, such as AD, may be associated with stroke (11). A large-scale meta-analysis showed that the risk of AD is 1.6 times higher in patients with ischemic stroke (12), and patients who have ischemic stroke are more prone to simultaneously develop AD (13). Chi et al. observed that Alzheimer’s patients are more vulnerable to intracerebral hemorrhage and ischemic stroke (14). This might be because of shared neuropathophysiological changes, including reduced blood flow to the brain, energy deficits, inflammation, capillary dysfunction, immune system failure, oxidative stress, and alterations in the expression of proteins such asβ-amyloid and tau (15). Therefore, memory-related diseases may play a significant role in the development and progression of stroke, and identifying key factors linking the two could provide theoretical support for stroke prediction.

Given the shared influencing factors between memory-related diseases and stroke, research suggests a potential association between the two conditions (12). To investigate this, we employed nationally representative data from the China Health and Retirement Longitudinal Study (CHARLS) to study the connection between diseases affecting memory and stroke, aiming to bring forward new perspectives for the clinical handling of stroke in terms of diagnosis, prevention, and treatment.

## 2 Materials and methods

### 2.1 Data collection

**Figure 1:**
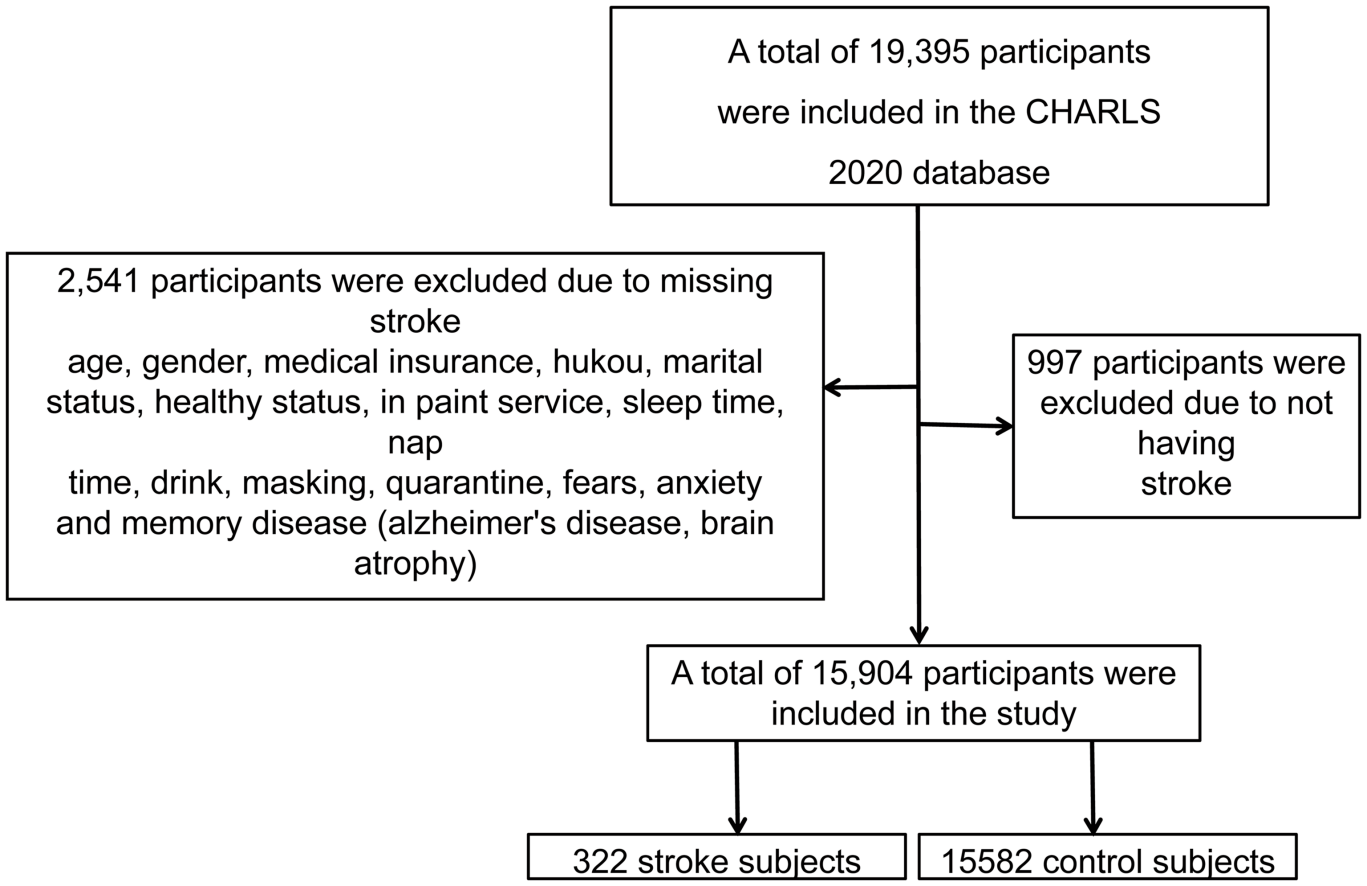
Flowchart of subject recruitment.

This study is a longitudinal study based on data from the CHARLS.^1^ In this study, a total of 19,395 subjects were collected from the CHARLS database in 2020 through a questionnaire-based approach. This study excluded 3,491 subjects with missing variables including age, gender, medical insurance, hukou, marital status, healthy status, in-patient service, sleep time, nap time, drink, masking during the pandemic, no quarantine experience, feeling fears during the Lunar New Year outbreak, feeling anxiety during the Lunar New Year outbreak and memory disease (AD, brain atrophy). Finally, a total of 15,904 participants were selected for this study, including 322 stroke subjects and 15,582 control subjects (Figure 1).

### 2.2 Assessment of stroke

In order to determine stroke, based on the dc003_8 questionnaire question: Has your doctor ever told you that you have any of these chronic conditions (Have you had a stroke?)? Subjects who answered “yes” to this question were considered to have a diagnosis of stroke as the disease group. Subjects who answered “no” to question served as the control group.

### 2.3 Exposure factors assessment

According to question da003_12 in the data section of the questionnaire: Has your doctor ever told you that you have memory disease (AD, brain atrophy)? Subjects who answered “yes” to this question were considered to have a diagnosis of memory disease as the disease group. Subjects who answered “no” to question da003_12 served as the control group.

### 2.4 Covariates

In addition, various covariates were also explored in this study, which included age (xrage), gender (ba001) (Male or Female), medical insurance (ba017) (Workers’, Social (urban rural), Social (urban), New Social (rural), Public or Other), hukou (ba009) (Agricultural, non-Agricultural or others), marital status (ba011) (Married with spouse present, Married but not living with spouse temporarily for reasons such as work, Separated, no longer living together as a spouse, Separated, no longer living together as a spouse, Widowed and Never married), healthy status (da001) (Good, Fair or Poor), in patient service (da007) (Yes or No), sleep time (da030) (>6 h or ≤6 h), nap time (da031) (>30 min or ≤30 min), drink (da051) (Yes or No), masking (va003) (Yes, No or Not go out), quarantine (vb005_s6) (Yes or No), fears (vc012) (Rarely never, Not often, Sometimes or Often times) or anxiety (vc013) (Rarely never, Not often, Sometimes or Often times) (16–18).

### 2.5 Analyses of statistical in participant characteristics

Based on the CHARLS database, all subjects were divided into two groups based on the presence or absence of stroke. There were differences in sample sizes among different covariates. When the sample size difference between the two groups was large, the results could be overly influenced by the characteristics of the large-sample group in a simple chi-square test. The influence of the two groups on the results could be balanced by a weighted chi-square test. Therefore, in this study, the differences in covariates of the baseline characteristics between the stroke group and the normal group of subjects were analyzed through a weighted chi-square test (*p* < 0.05). First, the data of each baseline characteristic of the subjects in the two groups were organized into a contingency-table form. Then, the expected frequency of each cell was calculated according to the row sum and column sum of the contingency table. The formula was: $E\mathrm{ij}=\frac{\mathrm{Ri}\times \mathrm{Cj}}{N}$ , where E_ij_ was the expected frequency of the cell in the ith row and jth column, R_i_ was the total number of the ith row, Cj was the total number of the jth column, and N was the total sample size. Next, the weighted chi-square value was calculated. The formula was: $X_{w}^{2}=\sum \mathrm{wij}{\frac{\left(\mathrm{Oij}-\mathrm{Eij}\right)}{\mathrm{Eij}}}^{2}$ , where O_ij_ was the actual observed frequency of the cell in the ith row and jth column, and W_ij_ was the weight of the cell in the ith row and jth column.

### 2.6 Statistical analyses

In order to investigate association between memory disease and stroke, three models were built by survey (v 4.2.1) package^2^: Model I (without any adjustments): the relationship between memory diseases and stroke; Model II (minimally adjusted model): adjusted for gender and age (added on Model I); Model III (fully adjusted model): further adjusted for medical insurance, hukou, marital status, healthy status, in patient service, sleep time, nap time, drink, masking, quarantine, fears, anxiety based on Model II. The results of this study were reported as odds ratios (OR) and 95% confidence intervals (CI), the value of *p* < 0.05 was considered statistically significant.

Stroke is usually a binary variable. Logistic regression is specifically used to deal with binary dependent variables and can well describe the relationship between independent variables and binary dependent variables. The association between memory diseases and stroke was analyzed through weighted logistic regression to verify the stability of the association between memory diseases and stroke risk among different populations. First, the independent variables and the dependent variable were determined. The dependent variable was stroke, and the independent variables were various variables. Then, the glm() function was used to construct a logistic regression model. The OR, 95% CI, and the *p*-value were calculated to determine the relationship between the independent variables and the risk of stroke (*p* < 0.05). Finally, pROC package (v 1.18.0) (19) was used to plot receiver operating characteristic (ROCs) curves to demonstrated accuracy and reliability of the Model III.

## 3 Results

### 3.1 Differences in subjects’ baseline characteristics

This study included 322 subjects in the stroke group and 15,582 subjects in the control group. In this study, there was a significant difference between stroke and normal groups in a variety of clinical characteristics (*p* < 0.001), including age, healthy status, patient service, memory diseases, drinking, anxiety, and masking (*p* < 0.05) (Table 1). The results included 52 subjects who were both patients with memory diseases and stroke, 399 subjects who were memory diseases but not stroke. These results all showed that memory diseases still showed a highly significant effect on stroke (*p* < 0.001).

**Table 1:** Differences in subjects’ baseline characteristics.

Basic Information	Level	Control	Stroke	*p*
*n*		15,582	322	
Hukou (%)	Agricultural	11,603 (74.5)	252 (78.3)	0.058
	Non-agricultural	2,321 (14.9)	49 (15.2)	
	Unified	1,658 (10.6)	21 (6.5)	
Marital_status (%)	Married_Living	12,107 (77.7)	249 (77.3)	0.928
	Separated_other	3,475 (22.3)	73 (22.7)	
Medical_insurance (%)	Workers’	2,245 (14.4)	38 (11.8)	0.768
	Social (urban_rural)	1,494 (9.6)	32 (9.9)	
	Social (urban)	707 (4.5)	14 (4.3)	
	New_Social (rural)	10,762 (69.1)	229 (71.1)	
	Public	170 (1.1)	3 (0.9)	
	other	204 (1.3)	6 (1.9)	
Age (mean (SD))		60.58 (9.41)	64.65 (8.66)	<0.001
Healthy_status (%)	Good	4,017 (25.8)	24 (7.5)	<0.001
	Fair	8,010 (51.4)	109 (33.9)	
	Poor	3,555 (22.8)	189 (58.7)	
In_patient_service (%)	Yes	2,710 (17.4)	145 (45.0)	<0.001
	No	12,872 (82.6)	177 (55.0)	
Sleep_time (%)	>6	6,224 (39.9)	103 (32.0)	0.005
	≤6	9,358 (60.1)	219 (68.0)	
Nap_time (%)	>30	7,009 (45.0)	154 (47.8)	0.338
	≤30	8,573 (55.0)	168 (52.2)	
Drink (%)	Yes	5,819 (37.3)	89 (27.6)	<0.001
	No	9,763 (62.7)	233 (72.4)	
Masking (%)	Yes	14,111 (90.6)	276 (85.7)	0.013
	No	659 (4.2)	20 (6.2)	
	Not_go_out	812 (5.2)	26 (8.1)	
Quarantine (%)	Yes	316 (2.0)	7 (2.2)	1
	No	15,266 (98.0)	315 (97.8)	
Fears (%)	Rarely_never	9,056 (58.1)	189 (58.7)	0.118
	Not_often	1,548 (9.9)	28 (8.7)	
	Sometimes	2,361 (15.2)	38 (11.8)	
	Often_times	2,617 (16.8)	67 (20.8)	
Anxiety (%)	Rarely_never	9,612 (61.7)	181 (56.2)	0.012
	Not_often	1,641 (10.5)	33 (10.2)	
	Sometimes	2,421 (15.5)	49 (15.2)	
	Often_times	1,908 (12.2)	59 (18.3)	
Gender (%)	Male	7,278 (46.7)	154 (47.8)	0.733
	Female	8,304 (53.3)	168 (52.2)	
Memory_disease (%)	Yes	399 (2.6)	52 (16.1)	<0.001
	No	15,183 (97.4)	270 (83.9)	

### 3.2 Correlation between memory diseases and stroke risk

Based on the three models constructed in this study, memory diseases were significantly associated with stroke in all three models (Model I, OR = 7.33, *p* < 0.001, 95% CI = 5.31–9.94; Model II, OR = 6.08, *p* < 0.001, 95% CI = 4.38–8.29; Model III, OR = 3.84, *p* < 0.001, 95% CI = 2.73–5.30), which suggested that the effect on memory diseases for stroke was not significantly confounded by other covariates (Table 2).

**Table 2:** Analysis of the association between exposure factors and outcomes.

Exposure	Model1_OR (95%_CI)	Model2_OR (95%_CI)	Model3_OR (95%_CI)
Memory_disease	7.33e + 00 (5.31e + 00 − 9.94e + 00)	6.08e + 00 (4.38e + 00 − 8.29e + 00)	3.84e + 00 (2.73e + 00 − 5.3e+00)
*p*_value	1.06E − 35	1.12E − 28	9.23E − 14

OR, Odds ratio; CI, Confidence interval.

### 3.3 Risk stratification analysis and model validation

**Figure 2:**
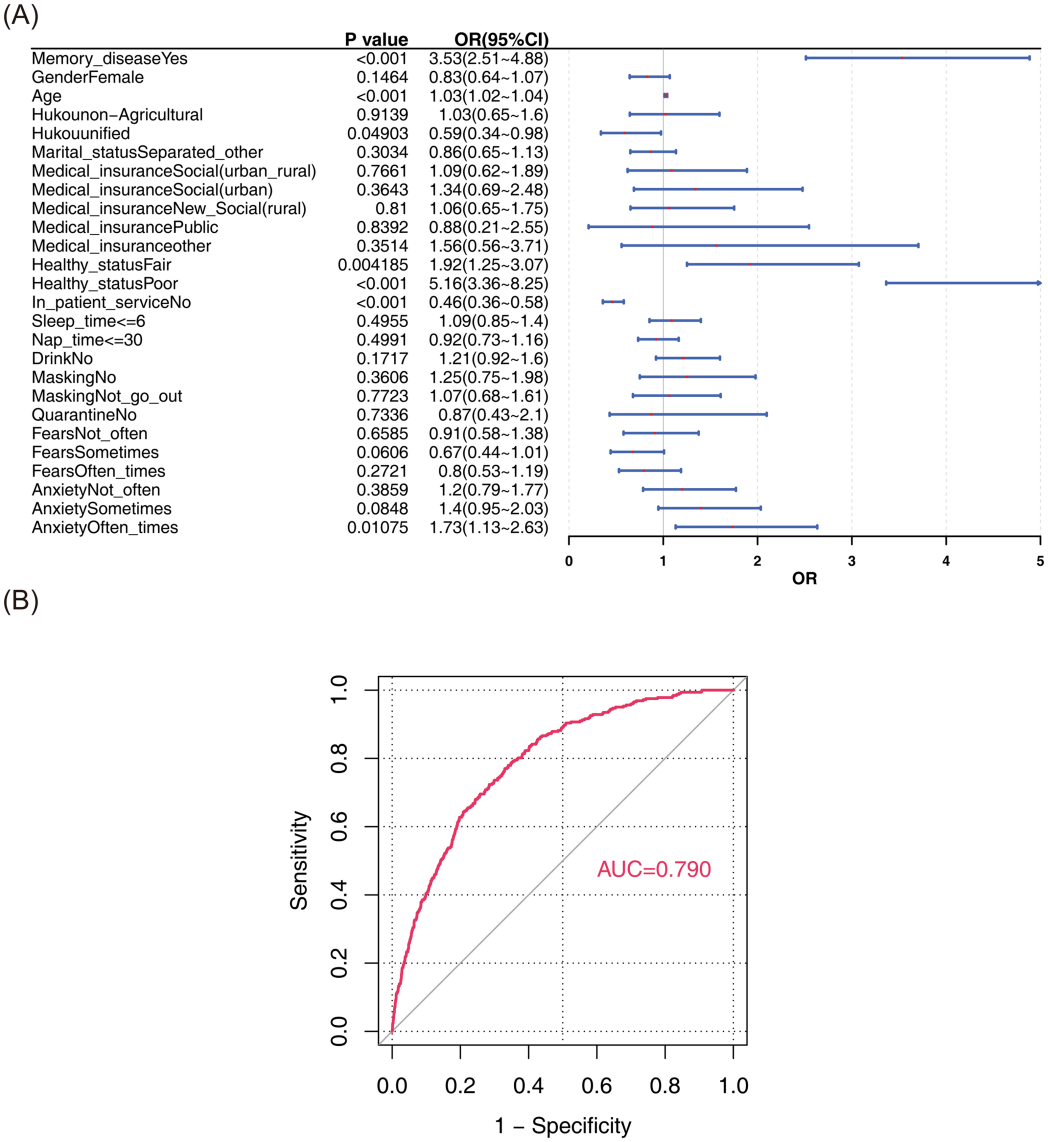
Risk stratification analysis and model validation. **(A)** Forest map. **(B)** ROC curve.

Weighted logistic regression analysis showed a strong association between memory diseases and stroke (Figure 2A), and memory diseases was a greater risk factor for stroke (OR = 3.53, *p* < 0.001, 95% CI = 2.51–4.88). Besides, the area under curve (AUC) of Model III was 0.790, which indicated that the prediction accuracy of Model III was better (Figure 2B).

## 4 Discussion

Stroke is characterized by a sudden cerebrovascular incident due to the rupture or occlusion of a brain blood vessel (20), which is characterized by high incidence, significant disability, and high mortality (21). Earlier studies have shown that stroke is linked to a range of factors, with AD thought to be highly related to hemorrhagic stroke, possibly because of shared neuropathophysiological changes (22). Therefore, the development and occurrence of stroke are closely related to diseases related to memory. This study, based on data from the CHARLS database, reveals a strong association between stroke and memory-related diseases, such as Alzheimer’s and brain atrophy. Memory-related diseases are significantly correlated with stroke, not only when considered as standalone exposure factors but also after adjusting for various covariates. These large-scale epidemiological data reveal a societal-level connection between memory-related diseases and stroke.

Our study has identified significant differences in a range of clinical characteristics between the stroke cohort and the control cohort, providing valuable insights for a more comprehensive understanding of potential risk factors and associations related to stroke. The observed age disparity between the stroke cohort and control group corroborates established epidemiological evidence, given that advancing age represents a well-documented non-modifiable risk factor for cerebrovascular events (23). Elderly individuals demonstrate increased susceptibility to vascular alterations such as atherosclerosis, which elevates the probability of both ischemic and hemorrhagic stroke occurrences (24). Notable differences in health status profiles between cohorts further underscore the critical role of systemic physiological conditions in stroke pathogenesis. Self-reported poorer health status may correlate with higher comorbidity burdens, particularly hypertension, diabetes mellitus, and cardiovascular disorders—all recognized principal stroke determinants (25). Disparities in healthcare utilization patterns warrant particular attention, with stroke-affected individuals demonstrating more frequent pre-morbid healthcare interactions, potentially indicative of pre-existing health concerns or differential healthcare accessibility (26). Lifestyle factors, notably alcohol consumption patterns, exhibited marked intergroup variation. Chronic excessive alcohol intake induces multiorgan damage, including hepatic (27) and cardiac impairments (28), with specific pathophysiological mechanisms involving endothelial dysfunction and prothrombotic states (29) that potentiate stroke risk. Alcohol-induced cirrhosis enhances hemorrhagic stroke susceptibility through coagulation profile alterations (30), while alcohol-related arrhythmogenesis (31), may promote intracardiac thrombus formation via hemodynamic disturbances (32), thereby increasing thromboembolic risks. In conclusion, this investigation identifies several clinically significant distinctions between stroke patients and non-stroke controls. These findings enhance our understanding of the intricate interplay between modifiable/non-modifiable factors and stroke vulnerability, while providing empirical evidence to inform targeted prevention strategies and optimized clinical interventions.

Our study found that stroke patients generally have poorer overall health compared to non-stroke individuals and often possess a history of recurrent hospitalizations. Previous studies support our findings. For instance, Sattam M et al. demonstrated that poor health significantly increases the risk of stroke, potentially due to the cumulative effects of chronic conditions such as diabetes, hypertension, and hypercholesterolemia, which impair the cerebrovascular system and elevate stroke risk (33, 34). Diabetes mellitus, fasting blood glucose levels between 110 and 125 mg/dL (6.1 to 6.9 mmol/L), and impaired glucose tolerance have been linked to a higher risk of stroke, with diabetes doubling the mortality risk in stroke patients (35, 36). Those with diabetes often face insulin resistance (37) and dyslipidemia (38), which include high LDL, elevated triglycerides, and low HDL cholesterol, all of which contribute to atherosclerosis and inflammation (39). Similarly, chronic high blood pressure might lead to atherosclerosis (40). These factors likely contribute to recurrent hospitalizations. From the perspective of disease associations, diabetes mellitus and hypertension serve as significant risk factors for both stroke and memory disorders, demonstrating complex interactions with these two conditions. However, in the current study, only one case had missing data for diabetes and hypertension, with all other samples exhibiting diseased status. This resulted in these two critical variables becoming constants in the model, precluding accurate evaluation of their confounding effects on the association between memory disorders and stroke. Future studies should increase sample sizes to ensure sufficient diversity in the prevalence of diabetes and hypertension. Concurrently, optimizing sample selection and allocation to better represent characteristics of diverse populations will enable precise assessment of the confounding effects of diabetes and hypertension on the stroke-memory disorder association.

This study undertook a comprehensive analysis of the relationship between memory-related diseases (Alzheimer’s disease and cerebral atrophy) and stroke. The findings demonstrated that across three models with varying degrees of adjustment, memory-related diseases were significantly associated with stroke. This association remained largely unaffected by other covariates, underscoring its substantial scientific and clinical relevance (41). In model 1, which included no adjustments, a strong association between memory-related disorders and stroke was observed. In model 2, the inclusion of age and gender as factors influenced the original association to some extent, suggesting that increased age and specific gender may serve as risk factors for both stroke and the onset and progression of memory-related diseases (42). Upon further adjustment for additional variables such as health status, alcohol consumption, mask-wearing, isolation, nervousness, and anxiety in model 3, the odds ratio (OR) was 3.84 (95% confidence interval: 2.73–5.30), which remained statistically significant (*p* < 0.05) despite a reduction in magnitude. This fully shows that even after considering many potential confounding factors, memory-related diseases are still a risk factor for stroke, and its impact on stroke occurrence is stable and independent (43).

More importantly, this study suggests that memory-related disorders such as Alzheimer’s disease (AD) and cerebral atrophy are risk factors for stroke. We hypothesize that this may be associated with shared pathophysiological mechanisms between the two conditions (12). Small vessel disease, characterized by white matter lesions and microinfarcts, is commonly observed in both disorders (44). These lesions disrupt neural connectivity, contributing to cognitive decline in AD patients and elevating the likelihood of stroke recurrence (45). Inflammation and oxidative stress play pivotal roles in both AD and stroke (46). Furthermore, the hallmark pathological features of AD—abnormal *β*-amyloid (Aβ) and tau protein deposition (47)—may also increase stroke risk by impairing cerebrovascular function (48). Our findings are consistent with multiple clinical studies reporting a risk association between memory impairment and stroke.

This study is subject to several limitations. Firstly, the research data were derived from self-reported questionnaires within the CHARLS database, rendering the diagnosis of stroke and memory-related diseases vulnerable to the accuracy of participants’ recall. This reliance on self-reporting increases the potential for recall bias and misclassification errors due to the lack of objective assessment metrics. Secondly, the diagnosis of brain atrophy was based solely on physician disclosure, without the support of quantitative imaging criteria, which complicates the distinction between physiological and pathological brain atrophy. Furthermore, the cross-sectional design of the study, despite adjustments for multiple variables, limits the ability to establish causal relationships between memory-related disorders and stroke. Additionally, unmeasured confounders, such as smoking, may have impacted the findings. Future research plans include expanding the sample size and conducting longitudinal studies, in collaboration with medical institutions, to obtain objective data such as medical records and imaging examinations, and to clarify stroke types and severity. This will be supplemented by long-term physiological monitoring using data from wearable devices. Furthermore, the integration of clinical manifestations, imaging findings, and biomarkers (such as beta-amyloid) enables precise differentiation of the pathological impacts of brain atrophy and Alzheimer’s disease (AD) on stroke, thereby clarifying their respective roles in stroke onset, progression, and prognosis. Additionally, efforts are underway to design prospective cohort studies and randomized controlled trials, develop multivariate regression models incorporating a broader spectrum of confounding variables (e.g., smoking), and investigate the efficacy of innovative preventive interventions for stroke among high-risk populations.

This investigation draws on the CHARLS, which includes 15,904 participants. A strong relationship between stroke and memory disorders, such as AD and brain atrophy, was confirmed in all three models. These conclusions suggest that further exploration of the mechanism between the two is justified and offer a new theoretical approach for diagnosing stroke diseases.

## Data Availability

The original contributions presented in the study are included in the article/supplementary material, further inquiries can be directed to the corresponding author.

## Footnotes

^1^ https://charls.pku.edu.cn/en/

^2^ https://cran.r-project.org/web//packages/survey/survey.pdf
